# The Effects of Exercise on Synaptic Plasticity in Individuals With Mild Cognitive Impairment: Protocol for a Pilot Intervention Study

**DOI:** 10.2196/50030

**Published:** 2023-10-18

**Authors:** Karishma R Ramdeo, Margaret Fahnestock, Martin Gibala, Ponnambalam Ravi Selvaganapathy, Justin Lee, Aimee Jennifer Nelson

**Affiliations:** 1 Department of Kinesiology McMaster University Hamilton, ON Canada; 2 Department of Psychiatry & Behavioural Neurosciences McMaster University Hamilton, ON Canada; 3 Department of Mechanical Engineering McMaster University Hamilton, ON Canada; 4 Department of Geriatric Medicine McMaster University Hamilton, ON Canada

**Keywords:** mild cognitive impairment, exercise, brain-derived neurotrophic factor, cognition, brain plasticity, repetitive transcranial magnetic stimulation, transcranial magnetic stimulation, magnetic stimulation, aging, interval training, intermittent theta-burst stimulation, repetitive transcranial magnetic stimulation, ageing, gerontology, geriatric, cognitive, physical activity, fitness, neurology, neuroscience, synapse, synaptic, plasticity, brain, neurotrophic, hormone, hormones, endocrinology

## Abstract

**Background:**

Mild cognitive impairment (MCI) is a syndrome preceding more severe impairment characterized by dementia. MCI affects an estimated 15% to 20% of people older than 65 years. Nonpharmacological interventions including exercise are recommended as part of overall MCI management based on the positive effects of exercise on cognitive performance. Interval training involves brief intermittent bouts of exercise interspersed with short recovery periods. This type of exercise promotes cognitive improvement and can be performed in individuals with MCI. Synaptic plasticity can be assessed in vivo by the neurophysiological response to repetitive transcranial magnetic stimulation (rTMS). A method to assess synaptic plasticity uses an intermittent theta burst stimulation (iTBS), which is a patterned form of rTMS. Individuals with MCI have decreased responses to iTBS, reflecting reduced synaptic plasticity. It is unknown whether interval training causes changes in synaptic plasticity in individuals living with MCI.

**Objective:**

This research will determine whether interval training performed using a cycle ergometer enhances synaptic plasticity in individuals with MCI. The three aims are to (1) quantify synaptic plasticity after interval training performed at a self-determined intensity in individuals with MCI; (2) determine whether changes in synaptic plasticity correlate with changes in serum brain-derived neurotrophic factor, osteocalcin, and cognition; and (3) assess participant compliance to the exercise schedule.

**Methods:**

24 individuals diagnosed with MCI will be recruited for assignment to 1 of the 2 equally sized groups: exercise and no exercise. The exercise group will perform exercise 3 times per week for 4 weeks. Synaptic plasticity will be measured before and following the 4-week intervention. At these time points, synaptic plasticity will be measured as the response to single-pulse TMS, reflected as the percent change in the average amplitude of 20 motor-evoked potentials before and after an iTBS rTMS protocol, which is used to induce synaptic plasticity. In addition, individuals will complete a battery of cognitive assessments and provide a blood sample from the antecubital vein to determine serum brain-derived neurotrophic factor and osteocalcin.

**Results:**

The study began in September 2023.

**Conclusions:**

The proposed research is the first to assess whether synaptic plasticity is enhanced after exercise training in individuals with MCI. If exercise does indeed modify synaptic plasticity, this will create a new avenue by which we can study and manipulate neural plasticity in these individuals.

**Trial Registration:**

ClinicalTrials.gov NCT05663918; https://clinicaltrials.gov/study/NCT05663918

**International Registered Report Identifier (IRRID):**

PRR1-10.2196/50030

## Introduction

Memory impairment has long been seen as a common consequence of aging, but it could be seen as mild cognitive impairment (MCI), a precursor of dementias such as Alzheimer disease. MCI is characterized by evidence of cognitive decline without a substantial disruption to completing everyday tasks [1,2]. Currently, 15% to 20% of people aged 65 or older are living with MCI [3]. About 10%-15% of individuals with MCI progress to dementia [3], exacerbating the concern for reduced social and functional capacities of such a large number of people and their increased rate of mortality [1,2].

Risk factors for MCI include older age, sedentary lifestyle, low number of years of education, and factors such as hypertension and obesity, various cardiovascular diseases, and neuropsychiatric conditions including depression and anxiety [1]. MCI is recognized as an intermediate phase between a healthy brain state and dementia; however, not all individuals diagnosed with MCI are certain to progress to dementia, and some may even revert to normal life [4]. The progression to dementia is often preceded by subtle difficulties in performing everyday activities [5] and can include diagnoses of subjective cognitive impairment [6], mild behavioral impairment [7,8], and psychiatric disturbances including anxiety [9,10], depression [11-13], and posttraumatic stress disorder [14,15]. Amidst ongoing challenges in developing disease-modifying drugs, nonpharmacological interventions including exercise are recommended as part of the overall MCI management [16] based on the positive effects of exercise on cognitive performance [17-23].

Exercise promotes cognitive improvement [24-32], and individuals living with MCI can participate in various forms of exercise [33,34]. Evidence also supports the notion that exercise interventions have a positive influence on different facets of cognitive function in MCI such as global cognitive functioning [30], immediate recall [22,26-28], delayed recall [26], and executive function [31,32]. However, not all studies report improvements in cognitive performance following exercise training [35-37]. Further, physical exercise may present challenges related to mobility and movement that limit the accessibility of the intervention to all individuals. Nonetheless, physical exercise is an affordable and alternative approach to alleviate symptoms of cognitive decline [17-22,38,39].

Interval training can involve intermittent bouts of exercise performed at either a prescribed or self-determined intensity, interspersed with short recovery periods [40,41]. The latter enables participants to determine their own pace that they deem appropriate and tolerable. They are typically encouraged to identify a pace that is physically challenging and to rate their effort level or perceived exertion. This type of training can be performed by participants with a wide age range who are both healthy and have conditions such as type 2 diabetes [42-44], coronary artery disease [45,46], and obesity [47]. Evidence that aerobic-based interval training can improve cognitive function [24-32] suggests that cognitive improvement could be induced by aerobic interval exercise via upregulation of pathways that promote synaptic plasticity.

Synaptic plasticity involves activity-induced changes in specific patterns of neural activity that alter the strength or efficacy of synaptic transmission, playing a considerable role in the acquisition of information and learning of new behaviors [48]. Synaptic plasticity is modeled by long-term potentiation (LTP) and long-term depression (LTD). LTP increases synaptic transmission as a consequence of high-frequency stimulation of excitatory synapses or the correlation of presynaptic activity and postsynaptic depolarization [48]. In contrast, LTD decreases the strength and efficacy of synaptic transmission [49]. A significant association has been found between LTP, LTD, and age-related cognitive decline. Animal models have shown that reductions in cognition due to age correlate with reductions in the LTP originating in the hippocampus [49].

In humans, synaptic plasticity, and more specifically, LTP-like effects can be assessed in vivo by delivery of transcranial magnetic stimulation (TMS) in protocols called intermittent theta burst stimulation (iTBS) and 5-Hz repetitive TMS (5-Hz rTMS). Both forms delivered over the motor cortex induce LTP-like effects as measured by short-term increases in the efficacy of the corticospinal pathway from cortex to muscle [50,51]. These effects are analogous to animal models of LTP since they are mediated by glutamate and require glutamate binding at *N*-methyl-D-aspartate (NMDA) receptors [51]. In humans, the decline in neuronal excitability and synaptic function seen with aging contributes to memory loss as well as sensory and motor deficits [52]. Thus, in humans, iTBS and 5-Hz rTMS are noninvasive tools to assess whether aging and MCI populations demonstrate synaptic plasticity and whether interventions such as exercise can enhance the magnitude of synaptic plasticity. Compared to age-matched controls, individuals with MCI demonstrate blunted synaptic plasticity as indicated by a reduced response to 5-Hz rTMS [53] and iTBS [54]. These effects are reflected as decreases in motor evoked potentials (MEP) when compared to healthy controls. Healthy controls demonstrate MEP facilitation, as expected, observed by an increase in MEP amplitude during the delivery of a 5-Hz train. In contrast, 5 Hz–induced MEP facilitation is not seen in individuals with MCI, suggesting deficits of glutamate pathways responsible for synaptic potentiation [53]. The delivery of iTBS has yielded similar results in patients with MCI, demonstrating a lack of MEP facilitation [54].

Brain-derived neurotrophic factor (BDNF) is a key regulator of processes crucial for cognition, particularly learning and memory [55-57]. The presynaptic release of BDNF influences the activation, trafficking, and expression of NMDA receptors as a result of enhanced calcium influx. This calcium influx is thought to allow for the release of BDNF at the postsynaptic terminal. Postsynaptic BDNF release is essential for presynaptic vesicle cycling, which increases synaptic plasticity, ultimately leading to improvements in cognitive functioning [58]. BDNF expression is correlated with cognitive function [57] and is consistently elevated following exercise in healthy older adults, suggesting that the rise in BDNF levels with long-term exercise may reduce mental deterioration in patients with MCI [55].

Exercise increases the secretion of hormones and other factors into the blood from organs such as skeletal muscle, bone, and liver [59-70]. Some of these are known to increase brain BDNF either directly or indirectly. A bone-derived hormone called osteocalcin (OCN), which plays a role in bone mineralization and glucose metabolism, increases following exercise [71-75] and increases BDNF transcription and the number of BDNF vesicles transported to the synapse [76,77]. OCN is carboxylated at 3 different residues and is found in the blood in both carboxylated and partially or fully decarboxylated forms. Acute exercise increases the partially or fully decarboxylated forms of OCN in serum [72-75].

The proposed research will determine whether interval training performed at a self-determined intensity will enhance synaptic plasticity in individuals with MCI. The three aims are to (1) quantify synaptic plasticity after interval training performed at a self-determined intensity in individuals with MCI; (2) determine whether changes in synaptic plasticity correlate with changes in serum BDNF, OCN, and with cognition; and (3) assess compliance to the exercise schedule.

## Methods

### Screening

To determine the eligibility of those who have reached out to the study team via advertisements, individuals will be contacted to schedule a phone interview. During the phone interview, individuals will be provided with the details of the study, and eligibility for TMS will be assessed via a TMS screening questionnaire. Individuals will also be asked whether they have been diagnosed by a health care professional or physician with “Mild Cognitive Impairment” or “Mild Neurocognitive Disorder?” If the individual answers “Yes,” then they will be asked to provide a written medical note to confirm a diagnosis of MCI at the first scheduled session. In addition, the individual’s eligibility to complete the exercise protocol will be assessed using the Canadian Society for Exercise Physiology Get Active Questionnaire (GAQ). If the individual has contraindications to the GAQ, the participant will be asked to contact their family doctor and acquire a medical note clearing them for participation. The parameters of the study, including the procedures and collection measures, will be explained verbally to the participant. If the participant agrees to participate in the study and has no contraindications to the GAQ and TMS screening questionnaire, a time will be arranged for the individual to undergo the experiment. Participants will provide informed written consent on the day of data collection prior to any testing, as well as verbal reaffirmation before each procedure. Researchers will determine the capacity to consent for all participants at the commencement of the study, using a modified University of California, San Diego Brief Assessment of Capacity to Consent questionnaire.

### Ethical Considerations

Approval to conduct this study was granted by the Hamilton Integrated Research Ethics Board (ID # 14938) in partnership with Hamilton Health Sciences and conformed to the Declaration of Helsinki. In addition, this study has been registered and approved as a registered clinical trial on clincialtrials.gov (NCT05663918) in December 2022, a World Health Organization (WHO) accredited trial registry.

### Power

This pilot study will recruit 24 participants aged 55-80 years old diagnosed with MCI. To determine this sample size, the following features were included in an a priori calculation using G*Power (Heinrich-Heine-Universität Düsseldorf), α=.05, Power (1-β)=0.9, and the effect size was calculated using Trebbastoni et al’s findings [53].

### Experimental Design

Individuals will be recruited for assignment to 1 of the 2 equally sized groups: exercise or no exercise. The exercise group will perform the exercise intervention. The no exercise group will serve as the control.

### Experimental Protocol

**Figure 1:**
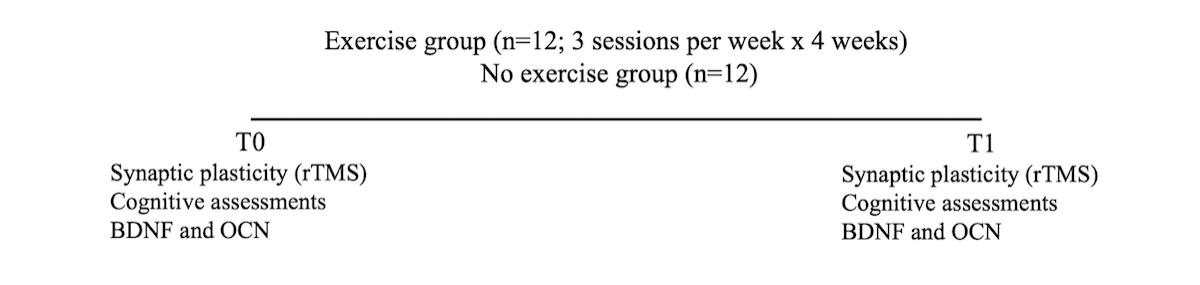
Dependent measures will be obtained in all participants 2-3 days before (T0) and following the exercise intervention (T1). Synaptic plasticity assessed using transcranial magnetic stimulation, cognitive function assessed by neuropsychological battery, and blood draws for serum BDNF and osteocalcin will be performed before and immediately following the intervention. BDNF: brain-derived neurotrophic factor; OCN: osteocalcin; rTMS: repetitive transcranial magnetic stimulation.

The dependent measures of synaptic plasticity, cognition, and venous blood BDNF and OCN, will be measured before and after the 4-week intervention (Figure 1).

### Exercise Intervention

**Figure 2:**
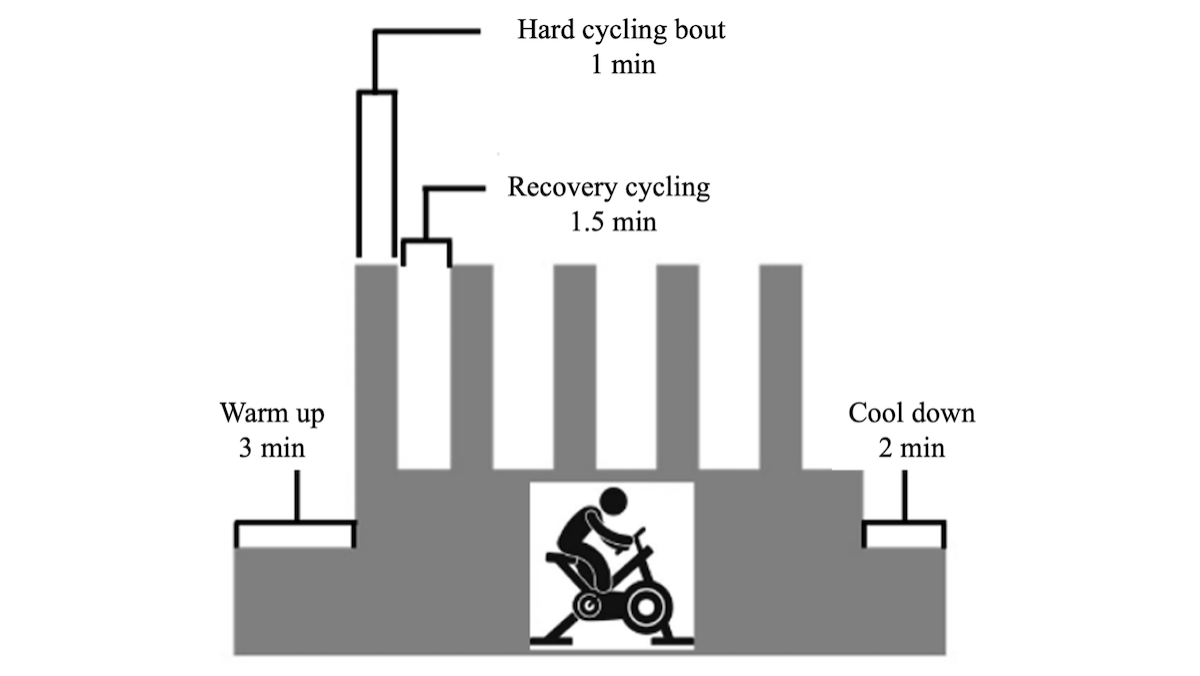
Self-determined intensity interval training protocol on cycle ergometer to be delivered to exercise group.

The exercise group will participate in 3 sessions per week of interval training performed at a self-determined intensity, using a cycle ergometer, for 4 weeks (12 sessions), in line with our previous experience in participant retention [41]. Ratings of perceived exertion (RPE) will be measured using Borg’s 6-20 scale [78]. Participants will be asked to exercise on a stationary bike at an intensity whereby their RPE is deemed “challenging” to them. The perceived effort and RPE rating will likely be different for participants depending on their initial fitness and exercise tolerance. The important aspect is for an individual to feel that the exercise is challenging. The cycling protocol includes 5 one-minute bouts of maximum effort, interspersed with 1.5 minutes of recovery, which will involve cycling at a light intensity (Figure 2). Participants will also perform a 3-minute warm-up and a 2-minute cool-down for a total exercise duration of 17.5 minutes, as we have described [41,74,79]. RPE will be measured by asking the participant to provide their rating at the end of the last interval.

### Dependent Measures

#### Synaptic Plasticity

**Figure 3:**
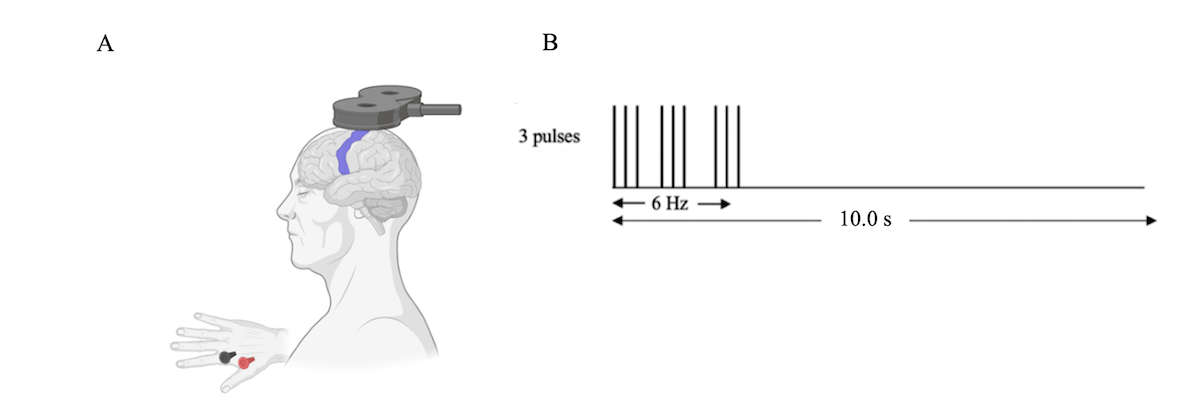
(A) Transcranial magnetic stimulation will be delivered over the primary motor area of the dominant hemisphere to find the optimal position for eliciting a motor evoked potential in the contralateral first dorsal interosseous muscle. (B) Intermittent theta burst stimulation parameters.

Surface electrodes (9 mm AgCl) will be used to record activity from the first dorsal interosseous (FDI) muscle of the right hand. The active electrode will be placed over the muscle belly. To reduce signal noise, dry ground will be placed on the styloid process at the right wrist. Electromyography (EMG) signals will be magnified ×1000 and bandpass filtered between 20 and 2.5 kHz (Intronix Technologies Corporation Model 2024F). An analog-digital converter will be used to digitize data at 5 kHz (Power1401; Cambridge Electronics Design), prior to being analyzed using commercial software (Signal version 7.01; Cambridge Electronics Design). Repetitive TMS will be performed using a 70-mm inner diameter figure-of-8 coil with a Magstim Super Rapid^2^ Plus Stimulator (Magstim). Biphasic magnetic pulses will be delivered over the primary motor area of the dominant hemisphere to find the optimal position for eliciting a MEP in the contralateral FDI muscle [80,81]. The hot spot of the right FDI muscle is defined as the location on the left motor cortex that, when stimulated with rTMS, consistently leads to the largest MEP in the muscle (Figure 3A). This point will be found and registered using Brainsight Neuronavigation and rTMS (Rogue Research).

Participants will complete 3 maximal isometric contractions of the right FDI against an immovable structure. The duration of each contraction will be 5 seconds with a 30-second rest interval between trials. The largest EMG activity obtained from any of the 3 trials will be defined as the maximum voluntary contraction (MVC) of the FDI muscle for an individual. The level of EMG activity corresponding to 10% MVC will be displayed on an oscilloscope as a horizontal target line. Participants will be required to match the horizontal target line and maintain 10% MVC by contracting their right FDI during the acquisition of active motor threshold (AMT).

AMT will be defined as the lowest intensity required to evoke a MEP≥200 μV in 5 out of 10 consecutive trials while participants maintain approximately 10% MVC with their FDI [80,81]. This value will be determined using TMS_MTAT_2.0 freeware [82]. The stimulus intensity will be set to 37% maximum stimulator output, and 20 TMS pulses will be distributed over M1, specifically the FDI hot spot, with the stimulus intensity being adjusted after each subsequent pulse as advised by the MTAT software based on the presence or absence of an MEP on the previous trial [83]. Resting motor threshold (RMT) will be defined as the lowest intensity required to evoke a MEP≥50 μV in 5 out of 10 consecutive trials while participants maintaining the FDI at rest [80,81]. This value will be determined using TMS_MTAT_2.0 freeware.

To assess synaptic plasticity, an iTBS protocol (Figure 3B) will be delivered using biphasic pulses in bursts of 3 pulses delivered in 6 Hz trains that will last 2 seconds, followed by 8 seconds with no pulse delivered [50,84,85]. iTBS will be repeated for a total of 612 pulses at 80% of AMT [86]. To assess synaptic plasticity 20 MEPs will be delivered at 120% of RMT. The average of 20 MEPs will be recorded from FDI before and immediately following iTBS.

#### Cognitive Function

Cognitive functions will be assessed using the National Alzheimer’s Coordinating Center Uniform Data Set Neuropsychological Battery, Version 3, comprised of the Montreal Cognitive Assessment, Semantic and Verbal Fluency, Trail-making Tests, Digit Span, Benson Complex Figure Test, and the Multilingual Naming Task.

#### Brain Derived Neurotropic Factor and Osteocalcin

Blood will be collected after ≥10-hour fast from an antecubital vein at T0 and T1 in red top BD Vacutainer collection tubes (Becton, Dickinson and Company, Franklin Lakes, NJ, USA). Red top tubes will be left to clot at room temperature for 45 minutes and subsequently centrifuged at 3488×g for 10 minutes at 4°C. Serum will be aliquoted into vials and stored at –80°C. Enzyme-linked immunosorbent assay (ELISA) assays will be carried out for the determination of serum BDNF (R&D Systems, Inc, Minneapolis, MN, USA) and total OCN (Human Osteocalcin ELISA kit KAQ1381). After incubation with 5 mg/ml hydroxylapatite (Invitrogen-Thermo Fisher Scientific, Carlsbad, CA, USA) to remove carboxylated OCN [87], serum uncarboxylated OCN will be measured by ELISA. Carboxylated OCN will be determined by subtracting uncarboxylated OCN from the total OCN [74].

#### Participant Experience

A Likert-type scale (0-4) will be given to participants to rate their enjoyment of the self-determined intensity interval training intervention (exercise group) and the overall research experience during the last visit (exercise and no exercise group).

#### Compliance

We will quantify the number of participants who demonstrate compliance with the exercise schedule. An individual is deemed 100% compliant if they attend all exercise sessions (3 times per week × 4 weeks=12 sessions). Attendance lower than this will be quantified as a percentage of the total number of sessions (out of 12).

### Data Exclusion

Peak-to-peak amplitudes of the MEPs will be collected by the experimenter. Trials will be examined visually, and any trials presenting with EMG activity prior to TMS stimulus artifact will be discarded from the analysis. The remaining EMG data will be analyzed using Signal Software (Cambridge Electronic Design).

### Statistical Analysis Plan

To investigate the difference in the magnitude of synaptic plasticity between groups, an unpaired *t* test will compare the percentage change in synaptic plasticity (from T0 to T1) between the 2 groups (exercise and no exercise). Similarly, the effect of exercise on serum BDNF and OCN levels will be assessed using an unpaired *t* test comparing the change in levels from T0 to T1. Significance will be set to =.05.

## Results

As of September 2023, we are currently screening participants for eligibility to participate in the research. We anticipate to complete data collection and analysis by August 2025.

## Discussion

### Overview

This pilot project is the first to assess changes in synaptic plasticity following a 4-week, self-determined, interval exercise training in individuals with MCI. Individuals will be assigned to 1 of the 2 groups (exercise or no exercise). The exercise group will participate in 12 sessions of exercise across 4 weeks (3 sessions per week). The no exercise group will not participate in the exercise regime, instead, they will be asked to return 4 weeks after their initial visit. Measures of synaptic plasticity, cognition, BDNF, and OCN will be assessed at baseline and 4 weeks later.

Previous research has demonstrated the positive benefits of exercise in individuals diagnosed with MCI. The 12 weeks of resistance training in individuals with MCI improved attention and working memory, and elicited changes in EEG activity [30]. In addition, 2 days of aerobic exercise in MCI subjects improved memory recall [26]. The 12 weeks of aerobic exercise in subjects with MCI improved cognition as reflected by changes in the Mini Mental State Exam [34]. These findings provide evidence that aerobic exercise is capable of modifying cognition in individuals with MCI. It remains unknown whether these improvements in cognition are a result of increases in synaptic plasticity following aerobic exercise.

Our study aims to determine whether synaptic plasticity is modified following 4 weeks of self-determined interval exercise training. Further, we will determine whether any changes in synaptic plasticity correlate with changes in cognition, BDNF, or OCN levels. We anticipate that the exercise group will demonstrate an increase in synaptic plasticity after 4 weeks of exercise compared to the no exercise group. If synaptic plasticity increases in the exercise group, this will be interpreted as a mechanism by which exercise improves cognition in MCI. Further, we hypothesize a positive relationship between a change in synaptic plasticity and measures of serum BDNF and OCN.

A limitation of this study is the use of self-determined interval training, whereby participants determine their own level of exertion. Individuals living with MCI will have varying physical and cognitive abilities which may limit their capacity to perform exercise at an imposed intensity. However, previous research has shown that only individuals who increase their fitness over the exercise period exhibit increased serum BDNF levels [88]. We hope that our approach of tailoring the exercise to the individual will increase the feasibility and retention of the study, thus creating a realistic and effective exercise regimen for individuals with MCI. A second limitation is that we do not consider biological sex or gender in the randomization due to the small sample size. If the study is successful in providing data to support compliance and the exercise regime, it will inform a larger-scale study of exercise training in MCI with biological sex and gender included.

### Conclusions

The data obtained from this research study will provide valuable insights into the compliance of a self-determined intensity interval exercise in the MCI population. Exercise provides an affordable and accessible opportunity to promote changes in cognition. This study will determine whether exercise alters synaptic plasticity in a population with impaired cognition.

## Abbreviations

AMT: active motor threshold
BDNF: brain-derived neurotrophic factor
ELISA: enzyme-linked immunosorbent assay
EMG: electromyography
FDI: first dorsal interosseous
GAQ: Get Active Questionnaire
iTBS: intermittent theta burst stimulation
LTD: long-term depression
LTP: long-term potentiation
MCI: mild cognitive impairment
MEP: motor evoked potential
MVC: maximum voluntary contraction
NMDA: N-methyl-D-aspartate
OCN: osteocalcin
RMT: resting motor threshold
RPE: rating of perceived exertion
rTMS: repetitive transcranial magnetic stimulation
TMS: transcranial magnetic stimulation
WHO: World Health Organization

## Data Availability

The data sets generated and analyzed during this study are available from the corresponding author on reasonable request.
